# Knockdown of PPAR δ Gene Promotes the Growth of Colon Cancer and Reduces the Sensitivity to Bevacizumab in Nude Mice Model

**DOI:** 10.1371/journal.pone.0060715

**Published:** 2013-04-08

**Authors:** Lie Yang, Jin Zhou, Qin Ma, Cun Wang, Keling Chen, Wenjian Meng, Yongyang Yu, Zongguang Zhou, Xiaofeng Sun

**Affiliations:** 1 Institute of Digestive Surgery and State Key Laboratory of Biotherapy, West China Hospital, Sichuan University, Chengdu, Sichuan Province, China; 2 Department of Oncology, Department of Clinical and Experiment Medicine, Linköping University, Linköping, Sweden; Mayo Clinic College of Medicine, United States of America

## Abstract

The role of peroxisome proliferator – activated receptor- δ (PPAR δ) gene in colon carcinogenesis remains highly controversial. Here, we established nude mice xenograft model using a human colon cancer cell line KM12C either with PPAR δ silenced or normal. The xenografts in PPAR δ-silenced group grew significantly larger and heavier with less differentiation, promoted cell proliferation, increased expression of vascular endothelial growth factor (VEGF) and similar apoptosis index compared with those of PPAR δ-normal group. After treated with the specific VEGF inhibitor bevacizumab, the capacities of growth and proliferation of xenografts were decreased in both groups while still significantly higher in PPAR δ-silenced group than in PPAR δ-normal group. Administration of PPAR δ agonist significantly decreased VEGF expression in PPAR δ-normal KM12C cells but not in PPAR δ-silenced cells. These findings demonstrate that, knockdown of PPAR δ promotes the growth of colon cancer by inducing less differentiation, accelerating the proliferation and VEGF expression of tumor cells in vivo, and reduces tumor sensitivity to bevacizumab. This study indicates that PPAR δ attenuates colon carcinogenesis.

## Introduction

Peroxisome proliferator – activated receptor-δ (PPAR δ), a member of the ligand-activated PPAR nuclear receptor family [1], is ubiquitously expressed in most tissues and highly expressed in epithelium, in particular skin and intestine [2], [3]. Similar to other nuclear hormone receptors, PPAR δ heterodimerizes with retinoid X receptor and exerts its effects via regulation of gene transcription upon binding of ligand [4]. The best-characterized role for PPAR δ to date is to regulate lipid metabolism and energy homeostasis, such as inducing reverse cholesterol transport, elevating high-density lipoprotein, increasing fatty acid oxidation and energy uncoupling [5], [6]. In addition, PPAR δ is also implicated in embryo implantation, wound healing, inflammatory response, endothelial cell proliferation, angiogenesis, skin cancer and colorectal carcinogenesis [7], [8].

In contrast to the well-characterized roles of PPAR δ in metabolic and energetic homeostasis, the role of PPAR δ in colorectal carcinogenesis remains uncertain. Some studies provide evidences that PPAR δ promotes tumorigenesis while others yield conflicting results, as we previously reviewed [9]. These inconsistent results dictate a need to further examine the function of PPAR δ in the pathogenesis of colorectal cancer. Recently, we successfully established the PPAR δ-knockdown models of colon cancer cell lines (KM12C etc.) by lentivirus-mediated RNA interfering (RNAi) [10]. We found that PPAR δ knockdown significantly induced less differentiation and promoted proliferation of these cells [9], [10]. These findings indicate that PPAR δ may play a tumor suppressor role by facilitating the differentiation and inhibiting the proliferation of colon cancer. However, there still lacks of in vivo experiment to testify these in vitro findings. To more rigorously define PPAR δ’s role in colorectal carcinogenesis, we examined the effect of PPAR δ knockdown on the nude mice xenografts established with KM12C cells in the present study.

## Materials and Methods

### Cell Culture

The human colon cancer cell line KM12C (from Professor IJ Fidler, Anderson Cancer Center, TX), untreated or treated by lentivirus-mediated RNA interfering (RNAi) against PPAR δ gene from our previous study [10], were used. Cells were maintained in Eagle’s minimal essential medium (MEM) with Earle’s salts, l-glutamine and nonessential amino acids (Sigma-Aldrich), supplemented with 1.5% NaHCO3, 1 mm Na-Pyruvate (Invitrogen, Carlsbad, CA, USA), 1×MEM Vitamin Solution (Invitrogen), 1% Penicillin-Streptomycin (Invitrogen), 1 µg/ml Puromycin (only for the treated cells to maintain the purity) and 10% fetal bovine serum (Invitrogen). The expression of PPAR δ have been stably silenced in the cells treated by lentivirus-mediated RNAi as examined in our previous study [10].

### Establishment of Tumor Xenografts in Nude Mice

Fourty-eight female nude mice of six weeks of age were purchased from the Sichuan University Laboratory Animal Center (Chengdu, China). Mice were maintained for 7 days in a conventional animal care unit before the start of the study.

Mice were anesthetized by intra-peritoneal injection of pentobarbital (65 mg/kg, Catalogue No.p3636, Sigma-Aldrich, Sweden). The KM12C cells either with stably silenced PPAR δ or untreated were then injected subcutaneously (2×10^6^ cells/mouse in 100 µl PBS) in the right flank of each mouse (24 mice per group). Twenty-four hours after inoculation, twelve randomly selected mice of each group were injected with bevacizumab (Avastin™, Genentech, CA) via tail vein at 5 mg/kg of body weight. Tumor growth was monitored at a regular interval by measuring two tumor diameters using electronic calipers. Tumor volume was calculated with the following formula: (length×width ^2^)/2. The mice were sacrificed 25 days after inoculation, and tumors were fixed in 10% neutral formalin. The procedures were operated in a blinded fashion by one investigator (L. Yang) without knowledge of grouping information.

Ethic statement: The animal handling was carried out in strict accordance with the recommendations in the Guide for the Care and Use of Laboratory Animals of the National Institutes of Health. The protocol was approved by the approved by the Animal Experimental Ethics Committee of Sichuan University. All surgery was performed under sodium pentobarbital anesthesia, and all efforts were made to minimize suffering.

### Hematoxylin and Eosin (HE) Staining

The xenografts from mice were regularly embedded in paraffinum and then sectioned at a thickness of 5 µm. The sections were deparaffinized in xylene, rehydrated in ethanol, rinsed in distilled water, and then fixed with 4% formaldehyde, stained with Ehrlich Hematoxylin and eosin (Sigma-Aldrich) followed by dehydration in graded alcohol. Slides were mounted and analyzed under light microscope.

### Immunohistochemical Assay (IHC)

The immunostaings were performed on 5 µm paraffin-embedded sections as previously reported [9]. The primary antibodies were rabbit polyclonal anti-PPAR δ IgG (ARP37889, Aviva Systems Biology, CA), anti-Ki67 Ig G (ab15580, Abcam, MA) and mouse monoclonal anti-VEGF IgG (ab68334, Abcam, MA). The Envision System Labelled Polymer-HRP Anti-Rabbit (Dakocytomation, CA) was used as a secondary antibody. Sections known to show positive staining for PPAR δ, Ki67 or VEGF were included in each run, receiving either the primary antibody or PBS, as positive or negative controls. In all staining procedures, the positive controls showed clear staining, whereas there was no staining in the negative controls.

The IHC slides were examined independently in a blinded fashion by two investigators (L.Y. and J.Z.) without knowledge of grouping information. The investigators scored each section by the staining intensity of tumor cells as follows: 0 (negative staining), 1 (weak staining exhibited as light yellow), 2 (moderate staining exhibited as yellow brown), and 3 (strong staining exhibited as brown). To avoid artificial effect, the cells on the margins of the sections and in areas with poor morphology were not counted. In the cases where the staining score had discrepant results, a consensus score was reached after re-evaluation.

### Cell Apoptosis Detection

The level of apoptosis was determined by terminal deoxynucleotidyl transferase (TdT)-mediated dUTP nick end-labeling (TUNEL) assay using the In Situ Cell Death Detection Kit (Catalogue No. 11684817910, Roche Diagnostics, Mannheim, Germany) following the manufacture’s protocol. Briefly, the sections were de-paraffinized, rehydrated, permeabilized and equilibrated. After labeling reaction, the results were evaluated using Leica DM IL HC fluorescent microscope. Total cells were visualized at 330–380 nm for DAPI staining, and apoptotic cells were visualized at 465–495 nm for FITC staining. Sections without adding TdT or receiving DNase I were included in each run as negative or positive controls respectively. For each sample, five high-power fields (×200) were randomly selected, and the number of apoptotic cells was counted for each field. Apoptosis index (AI) = number of positive cells/number of total cells.

### Real-time Reverse-transcription (RT) PCR

Total RNA was extracted from tumors using TurboCapture mRNA Kit (Qiagen, Germany), followed by reverse transcription with High-Capacity cDNA Reverse Transcription Kit (Applied Biosystems, CA). The mRNAs encoding VEGF, Ki67, adipocyte differentiation-related protein (ADRP), liver fatty acid binding protein (L-FABP), and intestinal alkaline phosphatase (ALPI) were quantified using real-time RT-PCR analysis by SYBR green detection. PCR reaction was performed on the Applied Biosystems 7500 Fast Real-Time PCR System, using comparative threshold cycle (Ct) method (△△Ct) as described before [11]. The primers used to quantify mRNAs were listed in Table 1. Expression was normalized to glyceraldehyde-3-phosphate dehydrogenase (GAPDH), and the 1× mix of GAPDH primers (Pre-developed TaqMan Assay Reagents, Applied Biosystems) was used for the detection. Each sample was analyzed in triplicate.

**Table 1:** Primers used to quantify mRNA.

mRNA	Primers
**VEGF**	(F) 5′-TACTGCTGTACCTCCACCTCCACCATG-3′
	(R) 5′-TCACTTCATGGGACTTCTGCTCT-3′
**Ki67**	(F) 5′-CGG ACT TTG GGT GCG ACT T-3′
	(R) 5′-GTC GAC CCC GCT CCT TTT-3′
**ADRP**	(F) 5′-CACAAATTGCGGTTGCCAAT-3′
	(R) 5′-ACTGGCAACAATCTCGGACGT-3′
**L-FABP**	(F) 5′-CCATGAACTTCTCCGGCAAGT-3′
	(R) 5′-TCCTTCCCTTTCTGGATGAGGT-3′
**ALPI**	(F) 5′-TGAGGGTGTGGCTTACCAG-3′
	(R) 5′-GATGGACGTGTAGGCTTTGCT-3′

VEGF, vascular endothelial growth factor; ADRP, adipocyte differentiation-related protein; L-FABP, liver fatty acid binding protein; ALPI, intestinal alkaline phosphatase; F, forward primer; R, reverse primer.

### Measurement of VEGF Production from KM12C Cells

VEGF production from KM12C cells was measured with a Human VEGF Colorimetric ELISA kit (Thermo Fisher Scientific Inc.,Rockford, IL). Briefly, KM12C cells (1×10^6^) with PPAR δ silenced or untreated were cultured in serum-free medium for 18 h. Then cells were treated with vehicle or indicated concentration of GW501516, the specific agonist of PPAR δ (0, 1, 2, 4, 8 µM) for 24 h. The supernatants were subjected to ELISA according to the manufacturer’s instructions. The color intensity of each well was determined on a microplate reader (Anthos htIII, Austria) at 450 nm. Calibration curves were constructed for each assay by plotting absorbance value versus the concentration for each calibrator. The VEGF concentrations of samples were then read from the calibration curve and normalized to nanograms per 10^6^ cells.

### Statistical Analysis

Statistical significance was determined using either a t-test, or, where applicable, rank sum test, analysis of variance (ANOVA) or Chi-square test using SPSS 13.0 software. The relative expression of mRNA was analyzed using the software REST-XL^©^ (available at http://www.wzw.tum.de/gene-quantification/) based on ΔΔCt method [11]. *P*<0.05 was taken as the significance level. Data shown represent at least three replicates of each experiment performed in triplicate.

## Results

### Knockdown of PPAR δ Promoted Tumor Growth and Decreased the Sensitivity to Bevacizumab

**Figure 1:**
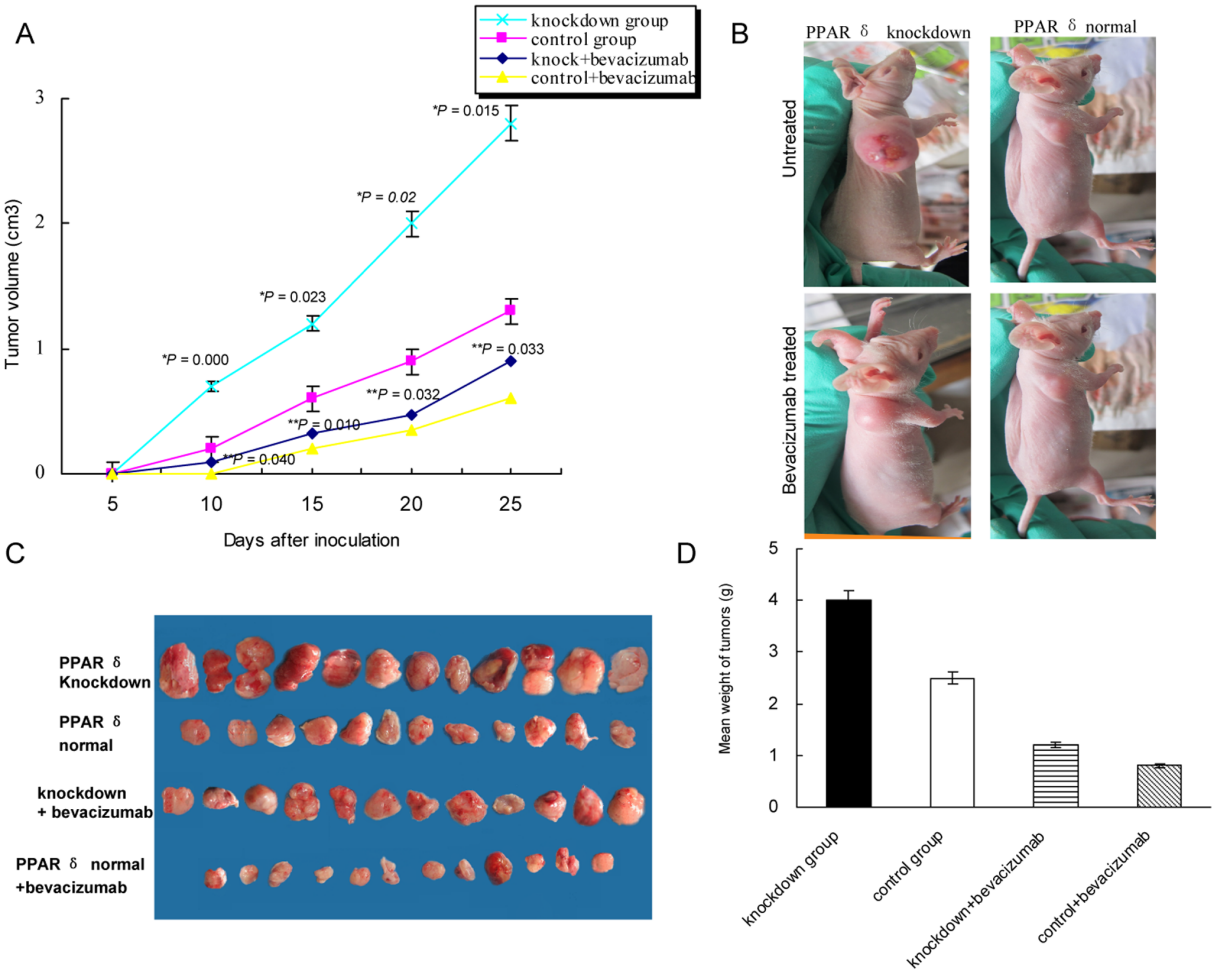
PPAR δ knockdown promoted the growth of xenografts in nude mice. (A). Growth curve of xenografts. At each time point, the xenografts in PPAR δ-silenced group (n = 12) grew significantly larger than those of control group (n = 12) (*P<0.05), even when treated by bevacizumab (**P<0.05) (mean±SD, t-test). (B). Representative photograph of mice in each group was taken 25 days after inoculation. (C). The xenografts when nude mice sacrificed. (D) The xenografts in PPAR δ-silenced group were significantly heavier than those in control group when nude mice sacrificed (mean±SD; *P = 0.021; **P = 0.02; t-test).

To examine the effect of PPAR δ knockdown on the tumor growth in vivo, we inoculated KM12C cells either with PPAR δ silenced or normal in nude mice and measured tumor volumes up to 25 days. As shown in Figure 1A, the slope of the xenograft growth curve was higher all along for PPAR δ-silenced group, and this difference in growth rates was statistically significant (*P*<0.05). At the end of the growth period, the mean tumor volume was 2.1 times higher for knockdown group than the control group (2.8 cm^3^ vs. 1.3 cm^3^, *P* = 0.015; Figure 1A–C), with an average weight of 4.0±1.4 g for knockdown group and 2.5±0.6 g for control (*P* = 0.021, Figure 1D).

To assess the relevance of combining PPAR δ knockdown with bevacizumab treatment, we injected bevacizumab to the mice. We found that, administration of bevacizumab significantly decreased the tumor growth of both groups, while the tumors in PPAR δ-silenced group grew significantly faster than PPAR δ-normal group (*P*<0.05; Figure 1A). The mean final volume of tumors was 1.4 times larger in knockdown group than the control group (0.9±0.11 cm^3^ vs. 0.6±0.15 cm^3^, *P* = 0.033; Figure 1A–C), with an average weight of 1.2±0.5 g for knockdown group and 0.8±0.2 g for control (*P* = 0.02, Figure 1D).

### The Xenografts with PPAR δ Knockdown were Less Differentiated

**Figure 2:**
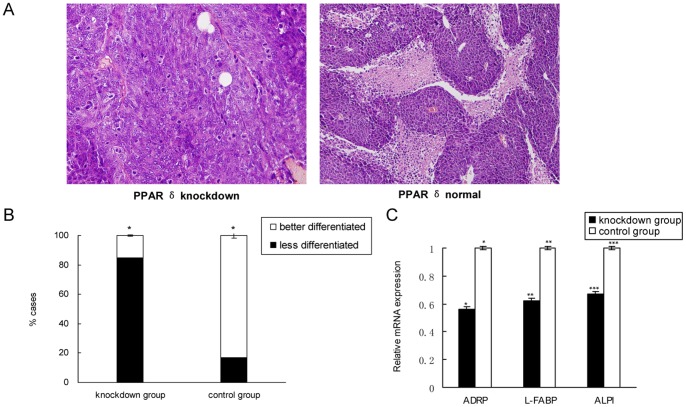
PPAR δ knockdown induced less differentiation of xenografts. (A). Representative photographs of xenografts by HE staining. The xenografts in PPAR δ-silenced group (n = 12) were obviously less-differentiated than those in control group (n = 12).×400 magnification. (B). PPAR δ- silenced group had significantly more less-differentiated xenografts than control group (85% vs. 17%, *P* = 0.025; Chi-square test). (C). Shown by quantitative RT-PCR, the mRNAs encoding ADRP, L-FABP or ALPI were significantly decreased in PPAR δ-silenced group relative to control group (*P*<0.05).

Following the Unified Standard of National Colorectal Cancer Pathology Research in China, the differentiation of tumor was graded with the percentage of adenoid structure components as follows: well-differentiated (>95%), moderately (50%–95%), poorly (5%–50%) and undifferentiated (<5%). By HE staining, we found significantly more less-differentiated (poorly+undifferentiated) xenografts in PPAR δ-silenced group than those in control group (85% vs. 17%, *P* = 0.025) (Figure 2A, B). We further quantified the genes associated with terminal differentiation, and found that the mRNAs encoding ADRP, L-FABP or ALPI were all significantly decreased in the PPAR δ-silenced group relative to the control group (*P*<0.05; Figure 2C).

### PPAR δ Knockdown Promoted the Proliferation of Tumor Cells

**Figure 3:**
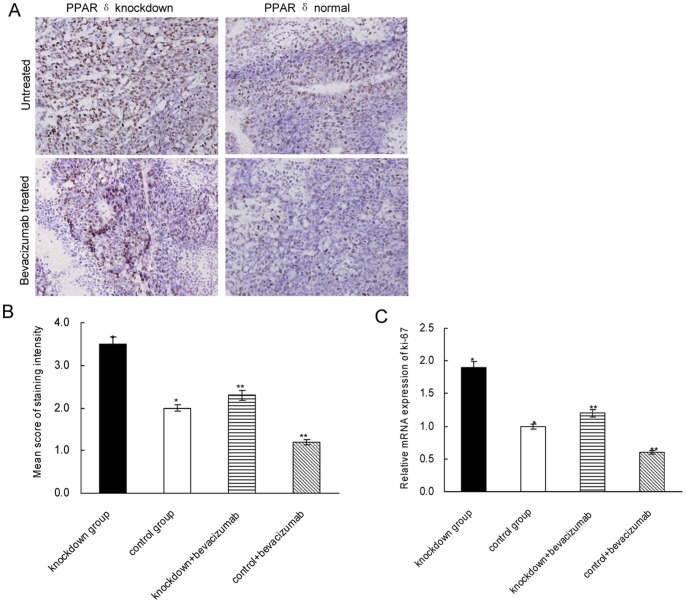
The expression of Ki67 in xenografts. (A). Representative photographs of Ki67 expression in xenografts stained by IHC.×400 magnification. (B). PPAR δ- silenced group (n = 12) had significantly higher score of Ki67 staining than control group (n = 12) (*P = 0.026), even after treated by bevacizumab (**P = 0.031) (mean±SD; t test). (C). The mRNA level of Ki67 was significantly higher in PPAR δ-silenced group than in control group (*P = 0.018), even after treated by bevacizumab (**P = 0.025).

We have previously shown that PPAR δ-silenced KM12C cells had a promoted growth rate in vitro [9]. To testify it in vivo, we examined the expression of the proliferation marker Ki67 in the xenografts. As shown by IHC assay, the PPAR δ-silenced group had significantly higher expression of Ki67 than PPAR δ-normal group (mean score: 3.5±0.4 vs. 2.0±0.6, *P* = 0.026; Figure 3A, B). After bevacizumab treatment, the expression of Ki67 was markedly decreased in both groups while still significantly higher in PPAR δ-silenced group than in the control group (mean score: 2.3±0.5 vs. 1.2±0.3, *P* = 0.031; Figure 3A, B). The quantitative RT-PCR yielded concordant result with the IHC assay (*P*<0.05; Figure 3C).

### PPAR δ Knockdown didn’t Affect the Apoptosis of Tumor Cells

**Figure 4:**
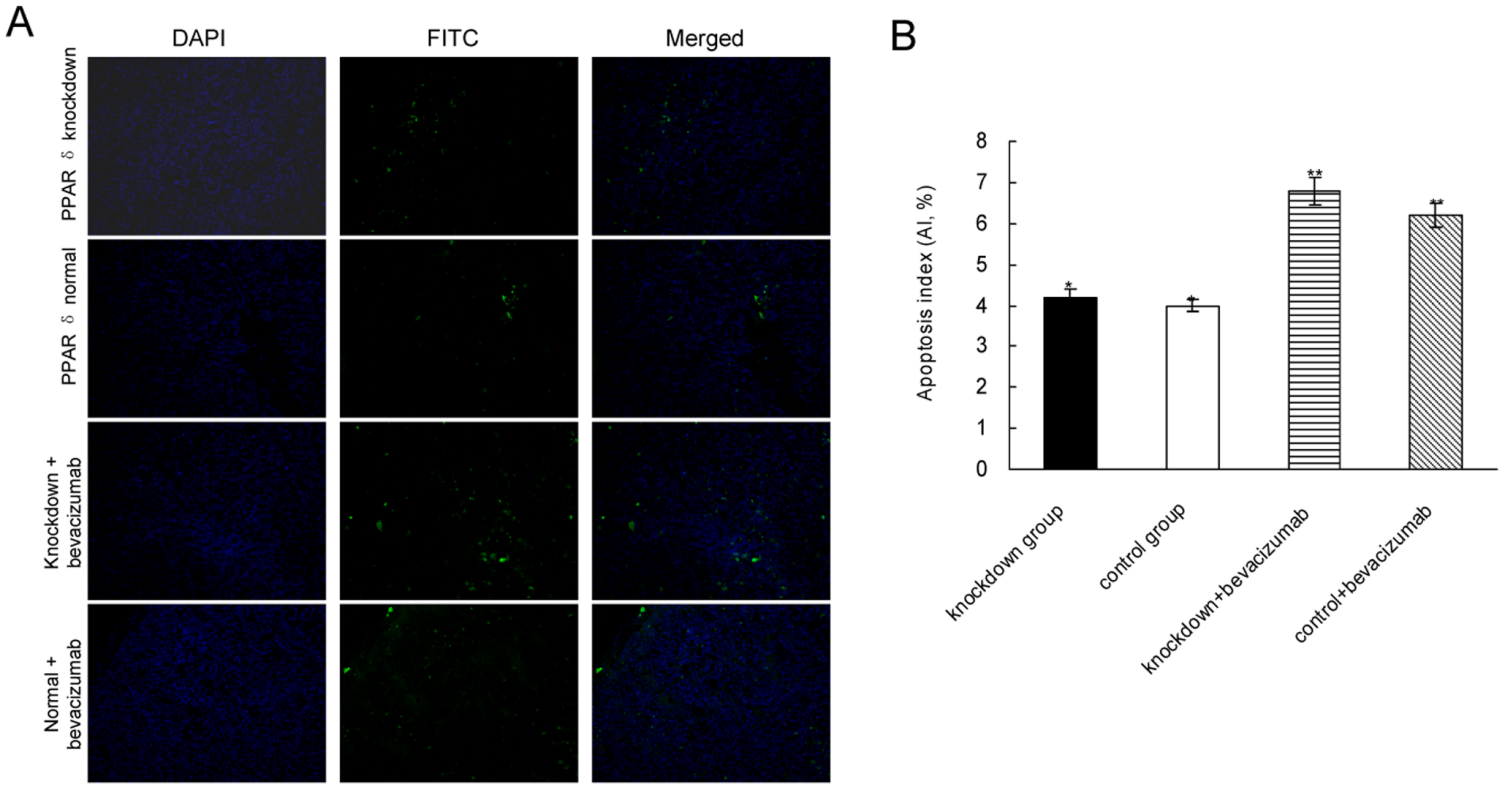
PPAR δ knockdown had no effect on apoptosis detected by TUNEL assay in xenografts. (A). Representative pictures of each group. The total cells were identified by DAPI stain, and the positive apoptosis cells were identified by FITC stain.×200 magnification. (B). Quantitative data of apoptosis index (AI). There wasn’t significant difference of AI between groups even after treated by bevacizumab (mean±SD,*P = 0.81, **P = 0.75; t-test).

The effect of PPAR δ knockdown on cell apoptosis was examined by TUNEL assay. As shown in Figure 4, significant difference of AI was not found between PPAR δ-silenced group and control group (6.2±4.7% vs. 7.0±3.6%, *P* = 0.81), even after bevacizumab treatment (10.8±4.1% vs.11.2±2.9%, *P* = 0.75).

### Knockdown of PPAR δ Induced VEGF Expression in Xenografts

**Figure 5:**
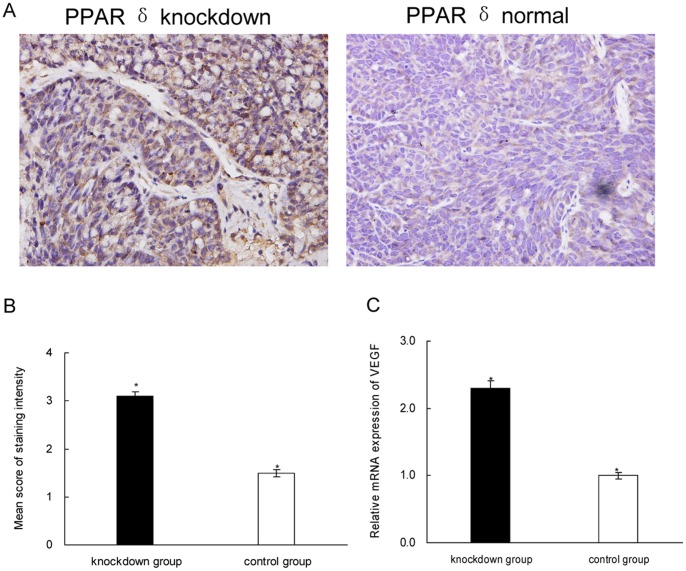
PPAR δ knockdown increased VEGF expression in xenografts. (A). Representative photographs of VEGF expression in xenografts stained by IHC.×400 magnification; (B). PPAR δ- silenced group (n = 12) had significantly higher score of VEGF staining than control group (n = 12) (*P = 0.028; rank sum test). (C). Quantitative RT-PCR result (*P = 0.032).

To analyze the correlation of PPAR δ with VEGF, we further examined the expression of VEGF in the xenografts. By IHC, we found that PPAR δ-silenced group had significantly more cases with high-expressed VEGF (score ≥2.0) (77% vs. 33%, *P* = 0.03) and higher intensity score than the control group (3.1±0.3 vs. 1.5±0.2, *P* = 0.028; Figure 5A, B). Quantitative RT-PCR analysis showed that, the mRNA of VEGF were significantly increased in PPAR δ-silenced group compared to the control (*P = *0.032; Figure 5C).

### Activation of PPAR δ Decreased VEGF Expression in KM 12C Cells

**Figure 6:**
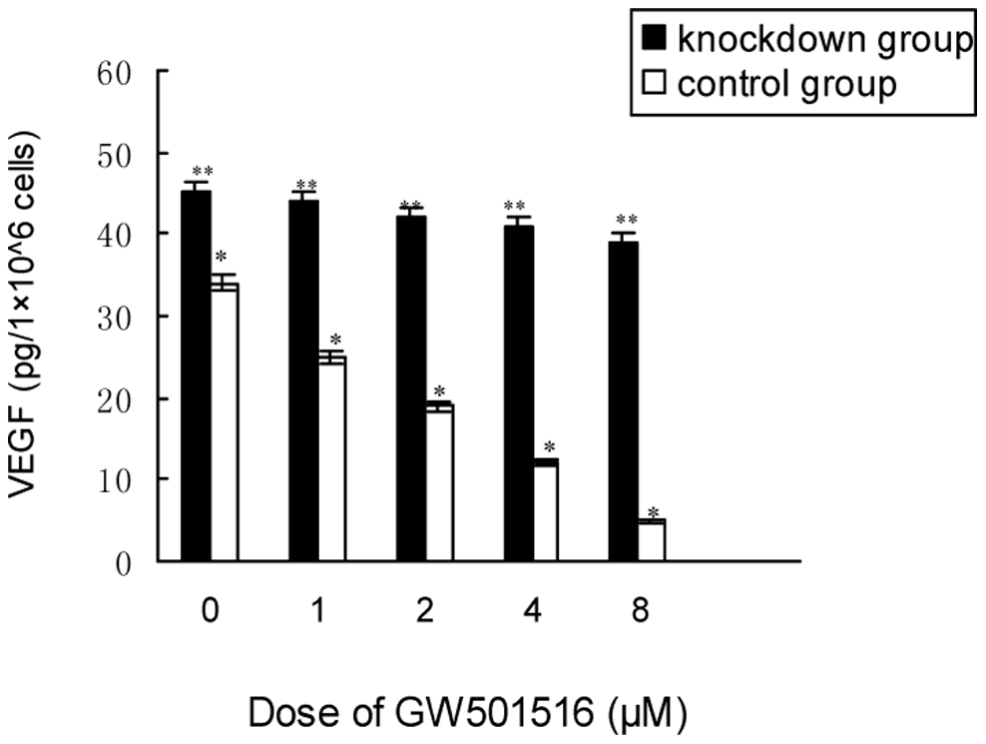
KM12C cells secreted less VEGF after PPAR δ activation. The KM12C cells were treated with serial concentrations of GW501516 or vehicle, and the cell-free supernatants were collected for VEGF quantification by ELISA. Treated by GW501516, the control cells showed a dose-dependent decrease of VEGF secretion (*P = 0.018), while the PPAR δ-silenced cells had no significant change all along (**P = 0.83; ANOVA).

To confirm the association between PPAR δ and VEGF, we treated KM12C cells with GW501516 (the specific agonist of PPAR δ) or vehicle, and quantified the VEGF in the cell-free supernatant by ELISA assay. As shown in Figure 6, the secretion of VEGF was much higher in the PPAR δ-silenced cells than in those with normal PPAR δ (control cells) before GW501516 administration (*P* = 0.035). After treatment with GW501516, the VEGF was decreased in a dose-dependent manner in the control cells (*P* = 0.018), while there wasn’t significant change in the PPAR δ-silenced cells all along (*P* = 0.83).

## Discussion

In the present study, we found that the xenografts in PPAR δ-silenced group grew significantly faster with less differentiation, promoted cell proliferation while similar apoptosis index and increased VEGF compared with those of PPAR δ-normal group, regardless of bevacizumab treatment. Administration of PPAR δ agonist significantly decreased VEGF expression in PPAR δ-normal KM12C cells, while didn't affect that of PPAR δ-silenced cells. These findings demonstrate that, knockdown of PPAR δ promotes the growth of colon cancer by lessening the differentiation and promoting the proliferation as well as VEGF expression of tumor cells in vivo, and reduces tumor sensitivity to bevacizumab. These results support a suppressor role of PPAR δ in the pathogenesis of colon cancer.

In the present study, our finding that the xenografts in mice grew significantly larger and heavier after PPAR δ knockdown indicates that PPAR δ may attenuate tumor growth in vivo. In accordance to this finding, recent studies show that the colon polyp formation was significantly greater in PPAR δ-deficient mice as compared with wild-type animals [12], [13]. In contrast to these observations, Park et al. [14] reported that PPAR δ-null HCT-116 cells had a decreased ability to form xenografts in nude mice. Another study concluded that PPAR δ is dispensable for polyp formation in the colon of APC^min^ mice [15]. However, these conclusions were based on the analysis of less than 6 mice, with limited statistical power. Result from the present study includes data from a total of 48 mice, providing more definitive evidence for a functional role for PPAR δ in colon carcinogenesis.

To clarify the mechanism underlying the promoted growth of tumors after PPARd knockdown, we further analyzed the differentiation, cell proliferation, apoptosis and VEGF expression in the xenografts. We found that the xenografts showed significantly less-differentiated histology after PPAR δ knockdown, with increased expression of differentiation-related genes ADRP, L-FABP and ALPI. These findings provide in vivo evidence that PPAR δ may facilitate the differentiation of colon cancer. This finding is consistent with recent studies which implicate PPAR δ in the regulation of epithelial differentiation: Activation of PPAR δ stimulates the terminal differentiation of keratinocyte [16]–[18]; PPAR δ promotes the differentiation of Paneth cells in intestinal crypts [19]. Recently, we show that PPAR δ knockdown induces less differentiation of colon cancer cell lines, and high expression of PPAR δ is related to better differentiation of rectal cancer [10]. These findings are consistent with the in vivo observations in the present study. The regulation on differentiation may underlie the promoting effect of PPAR δ knockdown on tumor growth as shown in this study.

The balance of proliferation and apoptosis plays a vital role in the control of tumor growth. The progression of tumor growth is characterized by increased proliferation and/or decreased apoptosis, or both. In the present study, we found that the expression of Ki67 was significantly increased while the apoptosis of tumor cells wasn’t changed after PPAR δ knockdown. It demonstrates that PPAR δ knockdown may promote the proliferation while have no effect on the apoptosis of colon cancer cells. This result is consistent with our previous observations in vitro, which show that PPAR δ knockdown promotes the proliferation of HCT-116 cells without effect on apoptosis [20]. The imbalance of proliferation and apoptosis is responsible for the promoted tumor growth after PPAR δ knockdown.

Angiogenesis is one of the main determinants of tumor growth, as tumor must stimulate the host to create its own vasculature to continue growing when it grows larger than 1–2 mm^3^ [21]. VEGF is a trigger of angiogenesis and essential for the development of blood vessels [22], [23]. Together with VEGF, other growth-related genes are involved in angiogenesis. A recent study showed that activation of PPAR δ up-regulated VEGF in colon cancer cells [24], implicating PPAR δ in the angiogenesis of colon cancer. In the present study, we show that VEGF was significantly increased in both the KM12C cells and the xenografts after PPAR δ knockdown, and decreased in PPAR δ-normal KM12C cells while unchanged in PPAR δ-silenced cells after treatment of GW501516. It demonstrates that activation of PPAR δ inhibits the expression of VEGF and thus may attenuate the angiogenesis of colon cancer. This result is consistent with the recent studies showing that PPAR δ may inhibit the proliferation of vascular endothelial cells [7], [25], [26]. The promotion of VEGF-mediated angiogenesis may be another factor underlying the promoted tumor growth after PPAR δ knockdown.

To examine the influence of PPAR δ knockdown on chemotherapeutic sensitivity, we treated the nude mice with bevacizumab. After the treatment, the tumor growth as well as cell proliferation was obviously slowed and the apoptosis was increased in both groups, while the PPAR δ-silenced group still showed higher capacities of tumor growth and cell proliferation than PPAR δ-normal group. This finding demonstrates that PPAR δ knockdown reduces the sensitivity of colon cancer to bevacizumab, underlying which may be the increased proliferation and lessen differentiation of tumors. It also implies that, VEGF mediated pathway is not the only mechanism by which PPAR δ regulates the tumor growth. To our knowledge, this is the first time to report the effect of PPAR δ on the chemosensitivity of colon cancer. It implies that, the colon cancer with normal or high expression of PPAR δ may have better response to bevacizumab than those with low expression of PPAR δ. Therefore, the expression level of PPAR δ might be a potential efficacy predictor of bevacizumab, and the development and application of PPAR δ-agonist agent may be a promising way to promote the efficacy of bevacizumab for colon cancer.

In conclusion, we show here that PPAR δ knockdown promotes the growth of colon cancer, inducing less differentiation and accelerating cell proliferation as well as VEGF expression, while has no effect on apoptosis, regardless of bevacizumab treatment. Ligand activation of PPAR δ decreases the expression of VEGF in colon cancer cells. These findings indicate that, PPAR δ may inhibit tumor growth by inducing differentiation, attenuating cell proliferation and VEGF-mediated angiogenesis in the pathogenesis of colon cancer, and facilitate the tumor sensitivity to bevacizumab. These results support the rationale for developing PPAR δ agonists for prevention and/or treatment of colon cancer.
